# Across days and levels: psychological richness and perceived social support in stress-related social inhibition

**DOI:** 10.3389/fpubh.2026.1945703

**Published:** 2026-10-28

**Authors:** Zhaoyan Meng, Mengyu Qu, Shisan Qi

**Affiliations:** 1 School of Psychology, Inner Mongolia Normal University, Hohhot, Inner Mongolia Autonomous Region, China; 2 Mental Health Education Research and Service Base, Key Research Base of Humanities and Social Science in Inner Mongolia Colleges and Universities, Hohhot, Inner Mongolia Autonomous Region, China

**Keywords:** daily stress, diary study, dynamic structural equation modeling, emerging adulthood, perceived social support, psychological richness, public mental health, social inhibition

## Abstract

**Background:**

Everyday stress and social inhibition are relevant to mental health during emerging adulthood. Daily psychological richness may help explain the temporal association between perceived stress and subsequent social inhibition.

**Methods:**

In a 15-day diary study, 186 emerging adults provided 2,790 observations of perceived stress, psychological richness, and social inhibition. Perceived social support was assessed at the person level. Two-level dynamic structural equation modeling (DSEM) was used to examine lagged associations and cross-level moderation.

**Results:**

Higher perceived stress was associated with lower psychological richness the following day [estimate = −0.588, 95% highest posterior density credible interval (HPD CrI) (−0.672, −0.501)]. Psychological richness was negatively associated with social inhibition on the subsequent day [estimate = −0.093, 95% HPD CrI (−0.108, −0.079)], and perceived stress showed a positive direct association with social inhibition 2 days later [estimate = 0.224, 95% HPD CrI (0.192, 0.257)]. Higher perceived social support was associated with weaker stress–richness and direct stress–inhibition associations. The credible interval for moderation of the richness–inhibition path included zero. The conditional indirect association was smaller at high than low support [difference = −0.040, 95% HPD CrI (−0.065, −0.016)].

**Conclusion:**

The findings identify a temporally ordered indirect association involving daily psychological richness and support-related differences in stress-linked pathways. They inform further research on everyday psychosocial experiences and social functioning among emerging adults.

## 1 Introduction

Emerging adulthood is a developmental period marked by expanding possibilities, role transitions, and heightened uncertainty. Typically spanning ages 18 to 25, this period involves major changes in education, work, identity, and interpersonal relationships (1). From a public mental health perspective, this life stage is important because stress, social functioning, and access to psychosocial resources can shape later patterns of adaptation. The World Health Organization has emphasized that mental health promotion requires attention not only to clinical services but also to everyday environments and protective resources that support well-being and participation (2). Emerging adults often report elevated stress, and perceived stress is associated with mental health, social functioning, and behavioral adjustment (3, 4). Perceived stress refers to the extent to which individuals appraise situations in their lives as unpredictable, uncontrollable, and overloaded (5). Compared with objective exposure to stressors, subjective stress appraisal may be a more proximal predictor of emotional, cognitive, and social outcomes (6–8).

Stress reflects a dynamic person-environment transaction. The transactional model of stress and coping emphasizes that appraisals and responses vary with daily changes in emotion, cognition, and available resources (9). Accordingly, public mental health research benefits from designs and models that capture within-person change and the temporal ordering of daily associations. This issue is particularly relevant for social inhibition, a regulatory tendency to restrain self-expression and interpersonal behavior in social contexts (10). Although social inhibition may serve a short-term defensive function when individuals perceive evaluation, uncertainty, or threat, persistent inhibition can reduce social participation, weaken opportunities for support, and constrain adaptive social engagement (11).

Conservation of Resources (COR) theory provides a useful framework for linking daily stress to public mental health outcomes. COR theory proposes that individuals strive to obtain, maintain, and protect valued resources, including personal characteristics, energies, social support, and conditions that facilitate goal attainment (12, 13). Stress occurs when resources are threatened, lost, or insufficiently replenished. Under such conditions, individuals are motivated to prevent further loss, often prioritizing resource conservation over resource acquisition (14). Social inhibition can therefore be understood as a resource-protective response: by limiting social exposure and self-expression, individuals may reduce immediate social risk and conserve energy when they feel stressed or vulnerable (15, 16). Sustained social restraint may also limit opportunities for social connection and help-seeking.

Perceived stress may be associated with social inhibition through changes in psychological resources. The present study focuses on psychological richness as one such resource. Psychological richness has been conceptualized as a third pathway to a good life, distinct from happiness and meaning, and characterized by diversity, complexity, novelty, and perspective change (17). A psychologically rich life involves experiences that broaden attention, challenge existing assumptions, and foster cognitive complexity (18, 19). A 14-day diary study found higher daily psychological richness on atypical days and on days involving novel activities (20), providing empirical support for its assessment at the daily level. At the daily level, psychological richness can be reflected in interesting, novel, emotionally varied, and memorable experiences that contribute to the diversity of everyday life.

Psychological richness may be relevant to public mental health through its links with experiential breadth, flexible appraisal, and openness to uncertainty. Stress can narrow attention toward threat and risk, reduce cognitive flexibility, and promote rigid coping (21–23). On days with higher perceived stress, emerging adults may engage less readily with novelty, complex, or perspective-changing experiences, potentially experiencing lower psychological richness on the following day.

Lower psychological richness may, in turn, be associated with greater social inhibition. When individuals have fewer opportunities for varied and perspective-changing experiences, ambiguous social situations may be perceived as more threatening and less manageable, potentially encouraging interpersonal restraint. In a cross-sectional study of Chinese university students, Gu et al. (24) found that psychological richness statistically mediated the association between COVID-19-related stress and social inhibition. This finding provides an empirical basis for examining the temporal organization of these associations in everyday life. Building on evidence of day-to-day variation in psychological richness, the present study examines whether daily stress, psychological richness, and subsequent social inhibition are associated across successive days within individuals. Accordingly, we proposed the following hypothesis:

*Hypothesis 1 (H1)*: Daily perceived stress is indirectly associated with social inhibition 2 days later through psychological richness on the intervening day.

Stress responses are also shaped by external resources. Perceived social support refers to the belief that emotional, informational, and instrumental help is available from family members, friends, and significant others (25). Perceived support has relatively stable person-level qualities and can buffer stress-related psychological risk (26, 27). Within the COR framework, social support represents a key external resource because it can compensate for losses, strengthen perceived control, and facilitate recovery when stress threatens internal resources (14).

Social support is relevant to public mental health because it is available through families, peers, educational settings, and communities. Prior research has linked perceived support to weaker associations between stress and withdrawal-related outcomes (28) and to less next-day behavioral discomfort following daily stress (29). These findings are consistent with established stress-buffering and resilience perspectives (30–32). Individuals with greater perceived support may therefore show weaker associations between daily stress and subsequent social inhibition, together with smaller stress-related reductions in psychological richness.

A further question concerns whether perceived social support also changes the strength of the pathway from psychological richness to social inhibition. Relational regulation theory suggests that supportive relationships can regulate affect, cognition, and behavior through ordinary interactions and shared activities (33). Reassurance, interpersonal feedback, and shared activities may shape how daily psychological richness is associated with subsequent social inhibition. On this basis, the study tested the following cross-level moderation hypotheses:

*Hypothesis 2 (H2)*: Perceived social support moderates the longitudinal indirect pathway from daily perceived stress to daily social inhibition through daily psychological richness.

*Hypothesis 2a (H2a)*: Perceived social support weakens the positive association between daily perceived stress and daily social inhibition.

*Hypothesis 2b (H2b)*: Perceived social support weakens the negative association between daily perceived stress and daily psychological richness.

*Hypothesis 2c (H2c)*: Perceived social support moderates the association between daily psychological richness and daily social inhibition.

The present study integrates COR theory with intensive longitudinal modeling to examine the associations among daily perceived stress, psychological richness, and subsequent social inhibition, and the moderating role of perceived social support. A 15-day diary design enables the estimation of lagged within-person associations alongside between-person moderation using DSEM (34, 35). This approach provides evidence on psychosocial processes relevant to social functioning and public mental health among emerging adults. The theoretical model is shown in Figure 1.

**Figure 1:**
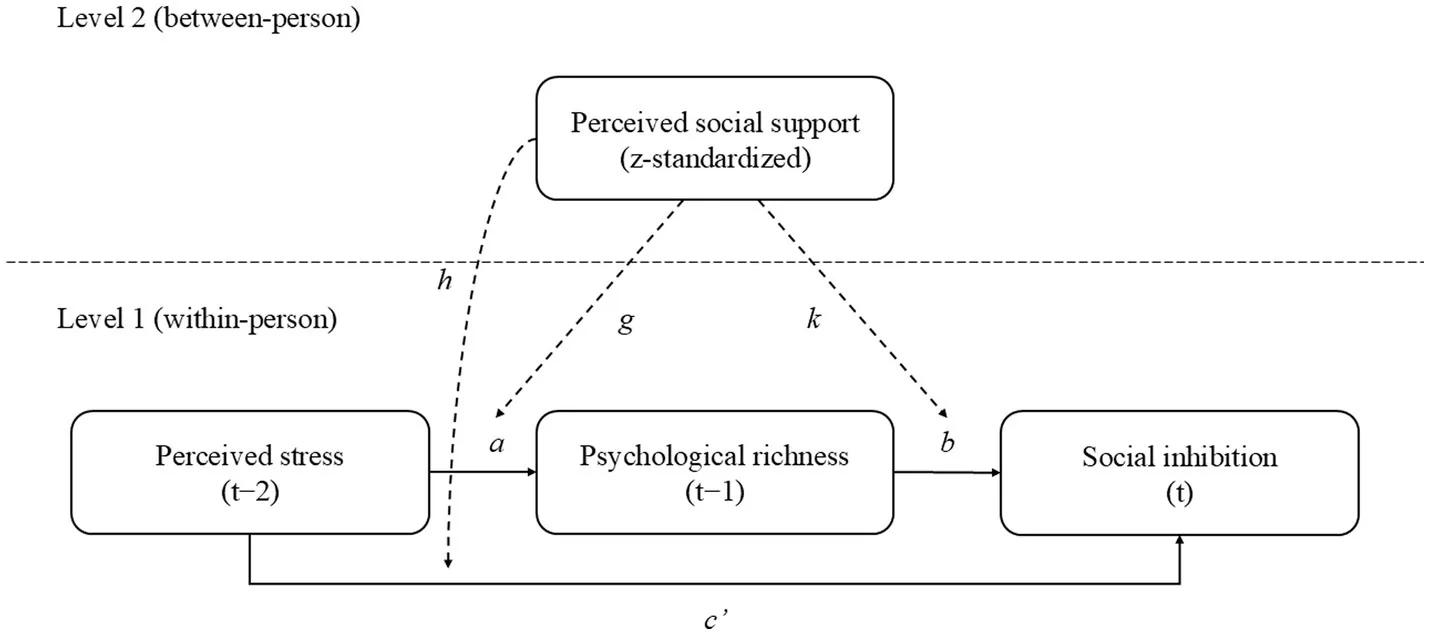
Temporal specification of the moderated 1-1-1 DSEM. The focal longitudinal indirect pathway is perceived stress (*t* − 2) → psychological richness (*t* − 1) → social inhibition (*t*). Perceived social support is a Level-2 moderator of the random *a*, *b*, and *c*’ slopes. Autoregressive effects for perceived stress, psychological richness, and social inhibition were estimated but are omitted for clarity.

## 2 Method

### 2.1 Participants and procedure

A total of 200 emerging adults aged 18–25 years were initially recruited through Tencent Questionnaire using a range of online recruitment channels. Interested individuals were invited to join a WeChat group created for the diary study. Each participant was assigned a unique identification number, which was used throughout the study to match responses across the 15 consecutive diary days.

Participants completed one diary survey per day for 15 consecutive days. A reminder to complete the daily survey was sent through the WeChat group at 21:30 each evening. Participants received RMB 1 for each completed daily survey. An additional RMB 2 bonus was provided to participants who completed all 15 diary assessments with responses meeting the study’s data-quality requirements.

Across the 200 initially enrolled participants, 3,000 diary assessments were scheduled and 2,910 were completed, corresponding to an overall diary completion rate of 97.0%. Data screening was conducted before the primary analyses. Thirteen participants were excluded because they missed between 3 and 11 of the 15 diary assessments; three of these participants also showed multiple unusually short completion times. One additional participant who completed all 15 assessments was excluded because of a highly repetitive response pattern across the daily social inhibition items. The final analytic sample therefore consisted of 186 participants, representing 93.0% of the initially enrolled sample. All retained participants completed all 15 diary assessments, yielding 2,790 observations. The final sample included 120 women (64.5%) and 66 men (35.5%). Participants had a mean age of 21.13 years (*SD* = 2.04; range = 18–24 years).

### 2.2 Measures

Daily assessments used Chinese-language items referenced to experiences during the current day with the qualifier “Today.” The established four-item Perceived Stress Scale was retained, while psychological richness and social inhibition were assessed using six-item adaptations of their respective source instruments. The retained items and their cross-sectional measurement characteristics are detailed in Supplementary Tables S5–S7.

#### 2.2.1 Perceived stress

Daily perceived stress was assessed using the established four-item Perceived Stress Scale (5), supported by psychometric research in Chinese samples (36). The items captured perceived unpredictability, uncontrollability, and difficulty coping with daily demands. Responses were made on a 5-point scale from 0 (never) to 4 (very often). Positively worded items were reverse-scored, and the four items were summed; higher scores indicated greater daily perceived stress. Within-person Cronbach’s alpha was 0.777 [95% participant-bootstrap CI (0.759, 0.793)].

#### 2.2.2 Social inhibition

Daily social inhibition was assessed using six items selected from the 15-item Social Inhibition Questionnaire (37), for which a Chinese-language adaptation has been published (38). Two items represented each of the three content domains: behavioral inhibition, interpersonal sensitivity, and social withdrawal. Responses were rated from 1 (not at all) to 4 (completely) and summed to obtain a broad social-inhibition composite; higher scores indicated greater daily social inhibition. Within-person Cronbach’s alpha was 0.609 [95% participant-bootstrap CI (0.576, 0.638)].

#### 2.2.3 Psychological richness

Daily psychological richness was assessed using six items adapted from the Psychologically Rich Life Questionnaire (39), including items drawn from a 12-item Chinese version previously examined in a Chinese university sample (40). The six retained items corresponded to Items 1–5 and 7 of the 12-item version and assessed psychological, experiential, and emotional richness together with interesting, novel, and memorable experiences. Items were rated on a 7-point scale from 1 (strongly disagree) to 7 (strongly agree). The responses were summed; higher scores indicated greater daily psychological richness. Within-person Cronbach’s alpha was 0.771 [95% participant-bootstrap CI (0.752, 0.787)].

#### 2.2.4 Perceived social support

Perceived social support was assessed once, at the person level, using the full 12-item Multidimensional Scale of Perceived Social Support (25), for which Chinese-language psychometric evidence is available (41). The scale covers perceived support from family, friends, and significant others. Items were rated on a 7-point scale from 1 (strongly disagree) to 7 (strongly agree) and summed, with higher scores indicating greater perceived support. Cronbach’s alpha for the total scale was 0.786; alpha values for the family, friends, and significant-other subscales were 0.721, 0.668, and 0.694, respectively. The total score was used as the Level-2 moderator in the primary analyses.

### 2.3 Data analysis

Data preparation, descriptive analyses, generalizability-theory reliability estimation, and HTMT analyses were conducted in R 4.5.3. The diary-oriented item-structure and temporal-sensitivity analyses were conducted in Python using NumPy, pandas, and SciPy. The two-level DSEM was estimated in Mplus 8.3. Daily perceived stress, psychological richness, and social inhibition scores were reconstructed directly from the item-level data by summing their corresponding items. Perceived social support was calculated as a 12-item person-level sum score. Because perceived social support was a time-invariant Level-2 variable, the score was standardized across the 186 participants before model estimation.

Descriptive statistics, intraclass correlation coefficients (ICCs), and within- and between-person correlations were examined before the primary DSEM analysis. ICCs were estimated from two-level models by partitioning the variance of each daily construct into within-person and between-person components.

Reliability of the diary measures was additionally evaluated using a generalizability-theory framework for intensive longitudinal data. Variance components were estimated using linear mixed-effects models. Reliability for detecting within-person change was quantified using *R*_C_, and reliability of person-level scores aggregated across the 15 fixed diary days was quantified using *R*_kF_. Construct distinctiveness was further examined using level-specific heterotrait–monotrait ratios (HTMT). Within-person HTMT estimates were calculated from person-mean-centered item responses for perceived stress, psychological richness, and social inhibition. Between-person HTMT estimates were calculated from participant-level item means and included perceived social support. Ninety-five percent bootstrap intervals were obtained using 5,000 participant-level resamples. Ninety-five percent percentile confidence intervals for the reliability coefficients were estimated using 1,000 participant-level bootstrap resamples, retaining each participant’s complete 15-day trajectory.

The within-person measurement properties of daily psychological richness were evaluated using person-mean-centered item responses. A one-factor minimum-residual exploratory factor analysis (MINRES) was fitted to the within-person item correlation matrix, and 95% confidence intervals were obtained from 500 participant-level bootstrap resamples. Factor retention was examined using 1,000 within-participant day permutations and 1,000 itemwise circular-shift permutations. Leave-one-day-out analyses and comparisons of days 1–7 versus days 8–15 assessed the stability of within-person reliability and item structure across diary occasions (Supplementary Tables S1–S4).

An additional cross-sectional questionnaire dataset contained 600 responses to the full PRLQ-12 and SIQ-15. After excluding 27 responses with completion times below 90 s and 25 responses that failed the attention check, 548 were retained. Item analysis and confirmatory factor analysis were conducted using these records. The abbreviated PRLQ-6 one-factor model and SIQ-6 one-factor and three-correlated-factor models were estimated using WLSMV for ordinal responses. Item selection and confirmatory analyses were based on the same sample (Supplementary Tables S5–S7).

The primary hypotheses were tested using two-level DSEM. Perceived stress, psychological richness, and social inhibition were specified as time-varying variables and were decomposed into within-person and between-person components within the DSEM framework. Perceived social support was specified as a time-invariant Level-2 predictor. The time variable was defined in one-day intervals using TINTERVAL = day (1).

At the within-person level, first-order autoregressive effects were estimated for perceived stress, psychological richness, and social inhibition. The three focal lagged associations were estimated as random slopes across participants: perceived stress at t − 1 predicting psychological richness at t (the *a* path), psychological richness at t − 1 predicting social inhibition at t (the *b* path), and perceived stress at t − 2 predicting social inhibition at t (the direct *c*’ path). Residual variances of the three daily variables were also allowed to vary across participants.

At the between-person level, perceived social support predicted the random *a*, *b*, and *c*’ slopes, corresponding to the moderation parameters *g*, *k*, and *h*, respectively. Perceived social support also predicted the between-person components of psychological richness and social inhibition. The between-person covariance between psychological richness and social inhibition and the covariance between the random *a* and *b* slopes were estimated.

The longitudinal indirect association was specified according to the temporal sequence perceived stress (t − 2) → psychological richness (t − 1) → social inhibition (t). The *a* path was estimated in the stationary DSEM as the lag-1 association from perceived stress at t − 1 to psychological richness at t. The same stationary parameter represents the preceding t − 2 → t − 1 transition in the longitudinal indirect sequence. The *b* path represented psychological richness at t − 1 predicting social inhibition at t, and *c*’ represented perceived stress at t − 2 predicting social inhibition at t.

For standardized perceived social support *Z*, the conditional indirect association was calculated as $\mathrm{IE}(Z)=(a+\mathrm{gZ})(b+\mathrm{kZ})+\mathrm{cov}(a_{j},b_{j})$ , where Cov(*a*_j_, *b*_j_) represents the covariance between the participant-specific random *a* and *b* slopes. Conditional indirect associations were evaluated at *Z* = −1, 0, and +1, corresponding to one standard deviation below the mean, the mean, and one standard deviation above the mean of perceived social support. The difference between the conditional indirect associations at *Z* = +1 and *Z* = −1 was used to evaluate moderated mediation.

Bayesian estimation was conducted using two Markov chain Monte Carlo (MCMC) chains with 80,000 fixed iterations per chain and a random seed of 20,261,006. The default Mplus first-half burn-in procedure was used, with a thinning interval of 1. Convergence was evaluated using the potential scale reduction factor (PSR), with values below 1.05 indicating satisfactory convergence. The same model was additionally estimated using 40,000 iterations as a sensitivity analysis. Statistical inference was based on posterior estimates and 95% highest posterior density credible intervals (HPD CrIs). Credible intervals excluding zero were interpreted as evidence that the corresponding parameter was credibly different from zero.

The final analytic dataset contained complete data for all 186 participants across all 15 diary days; therefore, the primary DSEM was estimated from 2,790 observations without missing daily observations.

## 3 Results

### 3.1 Measurement reliability and construct distinctiveness

Generalizability-theory analyses indicated within-person change reliability (*R*_C_) of 0.778 for perceived stress, 0.771 for psychological richness, and 0.609 for social inhibition. Reliability of person-level scores aggregated across the 15 diary days (*R*_kF_) ranged from 0.980 to 0.982. The corresponding bootstrap confidence intervals and within-person Cronbach’s alpha coefficients are reported in Supplementary Table S1.

Within-person MINRES analysis of psychological-richness items yielded positive factor loadings ranging from 0.578 to 0.621 (RMSR = 0.009). Permutation-based parallel analysis supported one predominant dimension, with the first observed eigenvalue of 2.796 exceeding the 95th-percentile reference value of 1.095. Leave-one-day-out analyses yielded alpha values of 0.766–0.774, indicating relatively stable measurement performance across diary occasions (Supplementary Tables S2–S4).

In the additional cross-sectional sample (*N* = 548), PRLQ-6 showed an interpretable one-factor solution (robust CFI = 0.986, robust RMSEA = 0.069). For SIQ-6, the three-correlated-factor model showed better comparative fit than the one-factor model (robust CFI = 0.981 vs. 0.781), with a robust RMSEA of 0.092 (Supplementary Tables S5–S7).

Level-specific HTMT estimates ranged from 0.145 to 0.195 within persons and from 0.527 to 0.777 between persons. The largest between-person value was observed for social inhibition and perceived social support [HTMT = 0.777, 95% bootstrap CI (0.697, 0.849)], supporting the distinguishability of the constructs assessed at each level.

### 3.2 Descriptive statistics and multilevel correlations

Descriptive statistics and level-specific correlations are presented in Table 1. At the within-person level, perceived stress was negatively correlated with psychological richness (*r* = −0.118) and positively correlated with social inhibition (*r* = 0.100), whereas psychological richness was negatively correlated with social inhibition (*r* = −0.133).

**Table 1:** Descriptive statistics and within- and between-person correlations.

Panel A. Descriptive statistics			
Variable	*M*	*SD*	Cronbach’s alpha
1. Perceived stress	11.189	3.995	0.777
2. Psychological richness	29.273	8.453	0.771
3. Social inhibition	19.409	3.383	0.609
4. Perceived social support	58.366	11.863	0.786

Panel B. Within-person correlations			
Variable	1	2	3
1. Perceived stress	—		
2. Psychological richness	−0.118***	—	
3. Social inhibition	0.100***	−0.133***	—

Panel C. Between-person correlations				
Variable	1	2	3	4
1. Perceived stress	—			
2. Psychological richness	−0.575***	—		
3. Social inhibition	0.714***	−0.699***	—	
4. Perceived social support	−0.482***	0.509***	−0.666***	—

Cronbach’s alpha represents within-person item consistency for perceived stress, psychological richness, and social inhibition, and person-level internal consistency for perceived social support. Within-person and between-person correlations were estimated from their corresponding covariance structures. Perceived social support was assessed at Level 2 only. ***p < 0.001.

At the between-person level, perceived stress was negatively correlated with psychological richness (*r* = −0.575) and perceived social support (*r* = −0.482), and positively correlated with social inhibition (*r* = 0.714). Psychological richness was negatively correlated with social inhibition (*r* = −0.699) and positively correlated with perceived social support (*r* = 0.509). Social inhibition was negatively correlated with perceived social support (*r* = −0.666). The differences between the within- and between-person correlation patterns further supported the separation of the two levels in the subsequent analyses.

### 3.3 DSEM

#### 3.3.1 Variance decomposition and model convergence

Variance decomposition indicated substantial variation at both the within-person and between-person levels. The ICC was 0.451 for perceived stress, 0.450 for psychological richness, and 0.556 for social inhibition (Table 2). Thus, 45.1%, 45.0%, and 55.6% of the total variance in the respective daily measures occurred between individuals.

**Table 2:** Within- and between-person variance components and intraclass correlations.

Variable	Within-person variance	Between-person variance	ICC
Perceived stress	8.765	7.190	0.451
Psychological richness	39.269	32.163	0.450
Social inhibition	5.076	6.369	0.556

ICC = between-person variance/(within-person variance + between-person variance). Variance components are reported on the summed-score metric used in the primary analyses.

The three daily variables also showed positive autoregressive associations. The posterior estimate for the lag-1 autoregressive effect was 0.569 for perceived stress [95% HPD CrI (0.506, 0.632)], 0.152 for psychological richness [95% HPD CrI (0.107, 0.195)], and 0.196 for social inhibition [95% HPD CrI (0.155, 0.238)], indicating persistence in each construct across adjacent diary days.

The final moderated DSEM showed satisfactory Bayesian convergence. With two MCMC chains and 80,000 iterations per chain, the highest final PSR was 1.003, below the prespecified criterion of 1.05. The corresponding 40,000-iteration sensitivity analysis produced virtually unchanged posterior estimates and credible intervals. All subsequent results are therefore based on the final 80,000-iteration model.

#### 3.3.2 Temporally aligned lagged paths and cross-level moderation

The focal posterior estimates are presented in Table 3. The stationary lag-1 association from perceived stress (t − 1) to psychological richness (t) was negative [estimate = −0.588, 95% HPD CrI (−0.672, −0.501)]. Under the stationarity assumption, the same coefficient represents the preceding perceived stress (t − 2) → psychological richness (t − 1) transition in the temporally aligned indirect sequence. Thus, higher-than-usual perceived stress was associated with lower psychological richness on the following day.

**Table 3:** Posterior estimates from the temporally aligned moderated 1-1-1 DSEM.

Path/effect	Posterior estimate	Posterior *SD*	95% HPD CrI
Within-person lagged paths			
*a*: Perceived stress (t − 2) → Psychological richness (t − 1)	−0.588	0.043	[−0.672, −0.501]
*b*: Psychological richness (t − 1) → Social inhibition (t)	−0.093	0.008	[−0.108, −0.079]
*c’*: Perceived stress (t − 2) → Social inhibition (t)	0.224	0.017	[0.192, 0.257]
Cross-level moderation by perceived social support			
*g*: Perceived social support → random *a* slope	0.299	0.045	[0.213, 0.388]
*k*: Perceived social support → random *b* slope	−0.013	0.007	[−0.028, 0.001]
*h*: Perceived social support → random *c’* slope	−0.066	0.016	[−0.097, −0.034]
Cov(*a*ⱼ, *b*ⱼ)	0.002	0.004	[−0.006, 0.010]
Conditional indirect effects			
Low support (−1 *SD*)	0.072	0.011	[0.052, 0.094]
Mean support (0 *SD*)	0.056	0.007	[0.043, 0.071]
High support (+1 *SD*)	0.032	0.008	[0.017, 0.049]
High − Low	−0.040	0.012	[−0.065, −0.016]

The a path was estimated as the stationary lag-1 association from perceived stress (t − 1) to psychological richness (t); under stationarity, the same parameter represents the t − 2 → t − 1 transition in the temporally aligned indirect sequence.

Psychological richness (t − 1) was negatively associated with social inhibition (t) [estimate = −0.093, 95% HPD CrI (−0.108, −0.079)], such that greater psychological richness was associated with lower subsequent social inhibition. The direct lag-2 association from perceived stress (t − 2) to social inhibition at t was positive [estimate = 0.224, 95% HPD CrI (0.192, 0.257)]. Together, these paths formed the temporally ordered sequence perceived stress (t − 2) → psychological richness (t − 1) → social inhibition (t), alongside the direct perceived stress (t − 2) → social inhibition (t) association.

Perceived social support moderated two of the three focal lagged associations. The moderation of the perceived stress → psychological richness path was positive [*g* = 0.299, 95% HPD CrI (0.213, 0.388)], indicating that the negative lagged association became weaker as perceived social support increased. At one standard deviation below the mean of perceived social support, the conditional *a* path was −0.887 [95% HPD CrI (−1.017, −0.759)]; at one standard deviation above the mean, it was −0.288 [95% HPD CrI (−0.404, −0.174)].

Perceived social support also moderated the direct lag-2 association between perceived stress and social inhibition [*h* = −0.066, 95% HPD CrI (−0.097, −0.034)]. The conditional direct association decreased from 0.291 [95% HPD CrI (0.243, 0.335)] at low perceived social support to 0.158 [95% HPD CrI (0.113, 0.203)] at high perceived social support.

The moderation of the psychological richness → social inhibition path was comparatively small [*k* = −0.013, 95% HPD CrI (−0.028, 0.001)]. The credible interval included zero, leaving H2c inconclusive.

#### 3.3.3 Conditional indirect effects

Conditional indirect associations were estimated from the complete moderated random-slope model, $\mathrm{IE}(Z)=(a+\mathrm{gZ})(b+\mathrm{kZ})+\mathrm{cov}(a_{j},b_{j})$ . The indirect association through psychological richness was credibly positive at each of the three prespecified levels of perceived social support and decreased as perceived social support increased. At low perceived social support (*Z* = −1), the conditional indirect association was 0.072 [95% HPD CrI (0.052, 0.094)]. At the mean (*Z* = 0), it was 0.056 [95% HPD CrI (0.043, 0.071)], and at high perceived social support (*Z* = +1), it was 0.032 [95% HPD CrI (0.017, 0.049)].

The posterior difference between the high- and low-support conditional indirect associations was −0.040 [95% HPD CrI (−0.065, −0.016)], indicating that the longitudinal indirect association was smaller at higher levels of perceived social support. The moderation parameter for the *b* path (*k*) had a credible interval that included zero, whereas the moderation of the *a* path (*g*) was credibly positive. The attenuation of the indirect association was therefore primarily associated with the weakening of the perceived stress → psychological richness pathway as perceived social support increased.

Figure 2 displays the posterior conditional indirect association and its 95% HPD credible interval across the observed range of perceived social support, with the conditional estimates at *Z* = −1, 0, and +1 highlighted.

**Figure 2:**
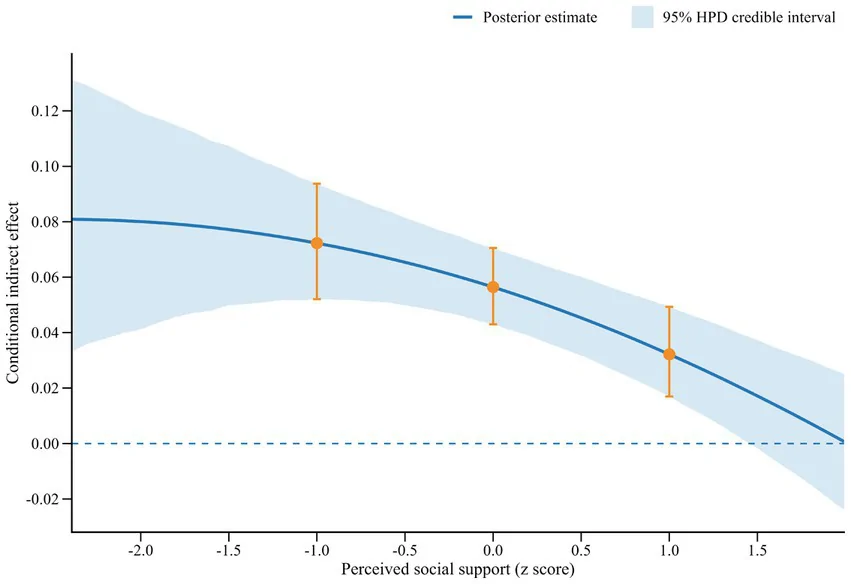
Conditional indirect effect of perceived stress on subsequent social inhibition through psychological richness as a function of perceived social support. The solid line represents the posterior estimate and the band represents the 95% HPD credible interval. The curve is shown only over the observed range of the standardized moderator (*z* = −2.391 to 1.992). Points indicate conditional indirect effects at −1 *SD*, the mean, and +1 *SD*.

## 4 Discussion

Using 15-day diary data and DSEM, this study identified a temporally ordered indirect association linking daily perceived stress to later social inhibition through psychological richness. A direct two-day stress–inhibition association also remained. Perceived social support was associated with weaker stress–richness and direct stress–inhibition paths, whereas evidence for moderation of the richness–inhibition path was inconclusive. These findings place daily psychological richness and person-level support at different points in the stress-related sequence of interpersonal restraint.

### 4.1 Psychological richness in the daily stress–inhibition sequence

Psychological richness has commonly been examined as a broader evaluation of life experience (17, 39). Gu et al. (24) reported a cross-sectional indirect association between COVID-19-related stress and social inhibition through psychological richness. In the present diary study, day-level variation in perceived stress was associated with psychological richness on the following day, which in turn was associated with social inhibition 1 day later. This sequence extends the evidence to temporally ordered within-person associations and is consistent with a resource-based account of stress (12, 13). The experiential and regulatory processes underlying these associations remain to be tested directly.

### 4.2 Perceived social support and differential buffering of lagged associations

The cross-level findings identify differences in lagged associations across levels of perceived social support. Participants reporting higher support showed a weaker negative stress–richness association and a weaker direct positive stress–inhibition association. These patterns are consistent with stress-buffering perspectives (30). The conditional indirect association was also smaller at higher support, although it remained positive. Because support was measured at the person level, these findings characterize differences between individuals in their within-person lagged associations.

The credible interval for moderation of the richness–inhibition path included zero, leaving H2c inconclusive. In contrast, the posterior difference between the high- and low-support indirect associations excluded zero. This pattern was driven primarily by moderation of the stress–richness path; moderation of the direct stress–inhibition path constituted a separate finding. The results therefore locate the clearest support-related differences in associations originating with daily stress. Studies with repeated support assessments or experimental manipulation could examine these patterns more directly.

### 4.3 Implications for mental health promotion and prevention

For public mental health research, the contribution lies in identifying a sequence of daily experiences that may be relevant to restrained interpersonal behavior among emerging adults. The results encourage attention to both the quality of everyday experiences and the perceived availability of support when examining stress-related social functioning. The distinction between a time-varying measure of psychological richness and a person-level support belief is particularly useful: it points to potentially different timescales at which internal experiences and social resources are associated with inhibition. Social inhibition was assessed through self-report, so the findings concern perceived interpersonal restraint rather than observed social participation, help-seeking, or clinical impairment.

These findings motivate experimental research on whether increasing everyday experiential variety, psychological richness, or perceived social support changes subsequent social inhibition under stress. Studies incorporating repeated resource assessments and behavioral measures of interpersonal interaction could clarify the mechanisms and practical relevance of these associations. In educational and community settings, such research could inform the development and evaluation of feasible approaches to supporting everyday social functioning.

### 4.4 Limitations and future directions

Several limitations qualify these interpretations.

First, the diary design established temporal ordering among measured variables, but the study was observational. The indirect pathway is a statistical association, and unmeasured time-varying influences may contribute to its component paths. The DSEM also assumed stationary lagged associations over the 15-day period and examined the specified one- and two-day intervals. Studies using experimental manipulations, reciprocal-path comparisons, alternative lag structures, or longer follow-up periods could test the proposed sequence and distinguish delayed associations from genuinely cumulative processes.

Second, perceived social support was assessed once as a person-level characteristic. Repeated measurement of perceived and received support would help distinguish stable differences in support beliefs from day-to-day support processes.

Third, the use of abbreviated self-report measures introduces measurement constraints. Social inhibition showed comparatively lower reliability for within-person change and was represented by a composite covering three related content domains. The psychological-richness items showed a predominant within-person dimension and relatively stable measurement performance across diary occasions. Future research incorporating formal longitudinal measurement-invariance tests, independent assessments of daily affect and curiosity, and behavioral indicators of social inhibition would strengthen the measurement evidence.

Finally, online recruitment and the complete-case analytic sample may have favored participants with greater adherence to the diary protocol. The available demographic information was limited to age and sex, restricting characterization of the sample’s educational, occupational, and geographical composition. Future studies could recruit more diverse participants and retain partially completed diary records to improve generalizability.

## 5 Conclusion

The 15-day diary findings identified a temporally ordered indirect association linking perceived stress to subsequent social inhibition through psychological richness on the intervening day. Perceived social support was associated with weaker stress–richness and direct stress–inhibition paths, alongside a smaller conditional indirect association; evidence for moderation of the richness–inhibition path remained inconclusive. These findings extend the study of psychological richness to daily social functioning and provide a basis for further experimental and longitudinal research on psychosocial resources in emerging adulthood.

## Data Availability

All data, analysis code, and research materials are available at https://osf.io/jdh6z/?view_only=d20d7725d99244f7a53233730d1afc38.
