# Supplementary material for: Across days and levels: psychological richness and perceived social support in stress-related social inhibition
Source: Front Public Health. 2026 Oct 28;14:1945703. doi: 10.3389/fpubh.2026.1945703 (PMC13751408; doi:10.3389/fpubh.2026.1945703)
Supplement: Supplementary file 1 [file Table_1.DOCX]

Supplementary Material

# 1 Reliability of daily within-person variation

Reliability of the three daily measures was assessed using within-person Cronbach’s alpha and generalizability-theory coefficients. R_C_ quantified the reliability of within-person change, and R_kF_ quantified the reliability of person-level aggregates across 15 days. Ninety-five percent percentile confidence intervals were obtained from 1,000 participant-level bootstrap resamples retaining complete diary trajectories (Table S1).

**Table S1**

*Reliability coefficients of the daily measures with participant-bootstrap confidence intervals*

| **Daily measure** | **Coefficient** | **Estimate** | **95% Bootstrap CI** |
| --- | --- | --- | --- |
| Perceived stress | R_C_ | 0.778 | [0.760, 0.794] |
|  | R_kF_ | 0.982 | [0.977, 0.986] |
|  | Within-person alpha | 0.777 | [0.759, 0.793] |
| Psychological richness | R_C_ | 0.771 | [0.753, 0.787] |
|  | R_kF_ | 0.982 | [0.977, 0.985] |
|  | Within-person alpha | 0.771 | [0.752, 0.787] |
| Social inhibition | R_C_ | 0.609 | [0.576, 0.638] |
|  | R_kF_ | 0.980 | [0.975, 0.983] |
|  | Within-person alpha | 0.609 | [0.576, 0.638] |

# 2 Exploratory within-person item structure

Each item was centered around its 15-day participant-specific mean. A one-factor minimum-residual exploratory factor analysis (MINRES) was estimated from the resulting within-person item correlation matrix. Factor-loading confidence intervals were computed by resampling participants (500 bootstrap replicates). Factor retention was evaluated using 1,000 permutations that independently shuffled each item’s 15 daily responses within participants. An additional 1,000 itemwise circular-shift permutations preserved each item’s within-person temporal order while disrupting cross-item day alignment.

The one-factor MINRES solution yielded six positive loadings ranging from 0.578 to 0.621 and a root mean squared off-diagonal residual (RMSR) of 0.009. Corresponding item-level estimates appear in Table S2. The first eigenvalue exceeded both permutation reference values; no subsequent eigenvalue did so (Table S3). These findings support a predominant common within-person dimension in the observed daily richness responses.

**Table S2**

*Within-person MINRES loadings and item–total relationships*

| Item | Loading | Bootstrap 95% CI | Corrected item–total r |
| --- | --- | --- | --- |
| M1 | 0.606 | [0.566, 0.638] | 0.520 |
| M2 | 0.621 | [0.589, 0.652] | 0.531 |
| M3 | 0.610 | [0.573, 0.645] | 0.523 |
| M4 | 0.583 | [0.548, 0.614] | 0.502 |
| M5 | 0.597 | [0.566, 0.631] | 0.513 |
| M6 | 0.578 | [0.542, 0.608] | 0.498 |

**Table S3**

*Within-person permutation parallel analysis*

| Dimension | Observed eigenvalue | Shuffled-days 95th percentile | Circular-shift 95th percentile |
| --- | --- | --- | --- |
| 1 | 2.796 | 1.095 | 1.099 |
| 2 | 0.673 | 1.054 | 1.059 |
| 3 | 0.664 | 1.026 | 1.028 |
| 4 | 0.648 | 1.003 | 1.003 |
| 5 | 0.616 | 0.982 | 0.980 |
| 6 | 0.602 | 0.956 | 0.951 |

# 3 Sensitivity across diary occasions

To evaluate dependence on particular diary occasions, reliability was recomputed after omitting each of the 15 days in turn and separately across days 1–7 and days 8–15. Item responses were re-centered within each participant for every analysis. These comparisons assess descriptive temporal robustness and results are summarized in Table S4.

**Table S4**

*Temporal-sensitivity results for psychological richness*

| Period or procedure | Within-person alpha | MINRES loading range | RMSR |
| --- | --- | --- | --- |
| All 15 days | 0.771 | 0.578–0.621 | 0.009 |
| Leave one day out (15 runs) | 0.766–0.774 | 0.571–0.628 | 0.007–0.011 |
| Days 1–7 | 0.759 | 0.567–0.605 | 0.014 |
| Days 8–15 | 0.749 | 0.526–0.602 | 0.012 |

*Note.* Leave-one-day-out ranges summarize the minimum and maximum estimates across 15 refits. For MINRES loadings, the range covers all six items across the 15 refits; it is not a confidence interval. All analyses used person-mean-centered item responses.

# 4 Additional cross-sectional item evaluation and abbreviated-scale structure

A separate cross-sectional questionnaire dataset contained 600 records with the 12-item psychological-richness measure (PRLQ-12) and the 15-item social-inhibition measure (SIQ-15). After application of response-duration and attention-check criteria (27 short-duration and 25 attention-check failures), 548 records remained. These analyses are separate from the 186-participant, 15-day diary dataset. The item-selection and confirmatory analyses in this section used the same 548 records and therefore do not constitute independent-sample validation or tests of longitudinal measurement invariance.

The six PRLQ items retained (Items 1–5 and 7 of the 12-item version) had the largest standardized loadings in an ordered-categorical one-factor analysis of the full item pool. For SIQ, two items per content domain were selected from the 15-item three-factor analysis: behavioral inhibition (SIQ1, SIQ4), interpersonal sensitivity (SIQ2, SIQ11), and social withdrawal (SIQ3, SIQ9). Table S5 reports the retained items with their original-pool estimates; Tables S6–S7 summarize the properties of the six-item scores.

**Table S5**

*Selected items and item-pool psychometric indices*

| Scale | Domain | Item | Pool CFA loading | Corrected item–total r |
| --- | --- | --- | --- | --- |
| PRLQ-12 | — | PRLQ1 | 0.846 | 0.761 |
| PRLQ-12 | — | PRLQ2 | 0.773 | 0.705 |
| PRLQ-12 | — | PRLQ3 | 0.819 | 0.729 |
| PRLQ-12 | — | PRLQ4 | 0.716 | 0.662 |
| PRLQ-12 | — | PRLQ5 | 0.695 | 0.630 |
| PRLQ-12 | — | PRLQ7 | 0.718 | 0.666 |
| SIQ-15 | Behavioral inhibition | SIQ1 | 0.889 | 0.644 |
| SIQ-15 | Behavioral inhibition | SIQ4 | 0.837 | 0.645 |
| SIQ-15 | Interpersonal sensitivity | SIQ2 | 0.850 | 0.686 |
| SIQ-15 | Interpersonal sensitivity | SIQ11 | 0.800 | 0.659 |
| SIQ-15 | Social withdrawal | SIQ3 | 0.815 | 0.658 |
| SIQ-15 | Social withdrawal | SIQ9 | 0.805 | 0.642 |

*Note.* PRLQ pool loadings derive from the 12-item one-factor CFA; SIQ pool loadings derive from the 15-item three-factor CFA. Corrected item-total r was calculated with the corresponding 12-item PRLQ total or five-item SIQ domain (not the shortened six-item score).

**Table S6**

*WLSMV confirmatory factor model fit for six-item measures*

| Scale | Model | Scaled *χ*² (df) | Robust CFI | Robust TLI | Robust RMSEA | SRMR |
| --- | --- | --- | --- | --- | --- | --- |
| PRLQ-6 | One factor | 33.717 (9) | 0.986 | 0.977 | 0.069 | 0.025 |
| SIQ-6 | One factor | 203.935 (9) | 0.781 | 0.636 | 0.255 | 0.092 |
| SIQ-6 | Three correlated factors | 30.167 (6) | 0.981 | 0.953 | 0.092 | 0.030 |

*Note.* Models were estimated using WLSMV for ordinal responses. *χ*² statistics are scaled; CFI, TLI, and RMSEA are robust indices. PRLQ-6 standardized loadings ranged from 0.698 to 0.850 (CR = 0.893, AVE = 0.583). The SIQ-6 three-correlated-factor model showed better comparative fit than its one-factor alternative, with robust RMSEA = 0.092 and two items per factor.

**Table S7**

*Reliability and domain coverage of six-item scores*

| Score | Retained items | Cronbach’s alpha |
| --- | --- | --- |
| PRLQ-6 | PRLQ1,2,3,4,5,7 | 0.880 |
| SIQ-6 | SIQ1,4,2,11,3,9 | 0.816 |
| SIQ-6 | SIQ1, SIQ4 | 0.786 |
| SIQ-6 | SIQ2, SIQ11 | 0.763 |
| SIQ-6 | SIQ3, SIQ9 | 0.709 |

*Note.* Observed domain-score correlations ranged from 0.459 to 0.490, and latent-factor correlations from 0.629 to 0.660.
